# The Scatman: an approximate method for fast wide-angle scattering simulations

**DOI:** 10.1107/S1600576722008068

**Published:** 2022-09-14

**Authors:** Alessandro Colombo, Julian Zimmermann, Bruno Langbehn, Thomas Möller, Christian Peltz, Katharina Sander, Björn Kruse, Paul Tümmler, Ingo Barke, Daniela Rupp, Thomas Fennel

**Affiliations:** a Laboratory for Solid State Physics, ETH Zürich, 8093 Zürich, Switzerland; b Institute for Optics and Atomic Physics, Technical University Berlin, 10623 Berlin, Germany; c Institute for Physics, University of Rostock, 18059 Rostock, Germany; d Department of Life, Light and Matter, University of Rostock, 18059 Rostock, Germany; DESY, Hamburg, Germany

**Keywords:** coherent diffraction imaging, wide-angle scattering, multi-slice Fourier transform, approximate methods, high-performance computing

## Abstract

Single-shot coherent diffraction imaging (CDI) is a powerful approach to characterize the structure and dynamics of isolated nanoscale objects such as single viruses, aerosols, nanocrystals and droplets. Using X-ray wavelengths, the diffraction images in CDI experiments usually cover only small scattering angles of a few degrees. These small-angle patterns represent the magnitude of the Fourier transform of the 2D projection of the sample’s electron density, which can be reconstructed efficiently but lacks any depth information. In cases where the diffracted signal can be measured up to scattering angles exceeding ∼10°, *i.e.* in the wide-angle regime, some 3D morphological information of the target is contained in a single-shot diffraction pattern. However, the extraction of the 3D structural information is no longer straightforward and defines the key challenge in wide-angle CDI. So far, the most convenient approach relies on iterative forward fitting of the scattering pattern using scattering simulations. Here the Scatman is presented, an approximate and fast numerical tool for the simulation and iterative fitting of wide-angle scattering images of isolated samples. Furthermore, the open-source software implementation of the Scatman algorithm, *PyScatman*, is published and described in detail. The Scatman approach, which has already been applied in previous work for forward-fitting-based shape retrieval, adopts the multi-slice Fourier transform method. The effects of optical properties are partially included, yielding quantitative results for small, isolated and weakly interacting samples. *PyScatman* is capable of computing wide-angle scattering patterns in a few milliseconds even on consumer-level computing hardware, potentially enabling new data analysis schemes for wide-angle coherent diffraction experiments.

## Abstract 2

A fast method for wide-angle coherent scattering simulations of weakly absorbing isolated samples, called the Scatman, is presented. Its quantitative agreement with exact solutions and the low simulation time of its software implementation *PyScatman* open new perspectives for single-shot 3D coherent diffraction imaging.

## 1. Introduction

Coherent diffraction imaging (CDI) aims to retrieve an isolated sample’s spatial information from the far-field amplitude of a highly coherent and monochromatic light beam that has scattered off the sample (Chapman & Nugent, 2010 ▸; Miao, Sandberg *et al.*, 2011 ▸; Seibert *et al.*, 2011 ▸). The great advantage of CDI is its lensless setup, making it suitable for those wavelength regions where lenses are hard or even impossible to manufacture. Thus, the spatial resolution in CDI is, in principle, only dependent on the radiation wavelength and on the maximum scattering angle at which the scattering signal can be recorded on a detector.

For small-angle scattering (SAS) conditions (Guinier *et al.*, 1955 ▸), and assuming the first Born approximation (Born, 1926 ▸), the 2D scattering image can be efficiently computed by calculating the squared absolute value of a Fourier transform (FT) of the imaged sample’s 2D electron-density projection. This relationship between the sample and the diffraction within the SAS regime is the basis of the original CDI approach, experimentally demonstrated for the first time in 1999 (Miao *et al.*, 1999 ▸), where iterative phase retrieval algorithms are employed to reconstruct the scattered field in the detector’s plane in amplitude and phase (Fienup, 1982 ▸; Marchesini, 2007 ▸). Upon successful phase recovery, the real-space 2D projection of the sample can be directly computed (Loh *et al.*, 2012 ▸; Seibert *et al.*, 2011 ▸; Pedersoli *et al.*, 2013 ▸).

It is still possible, under some specific circumstances, to perform 3D imaging in the SAS scheme, but the single-shot constraint has to be released. The most obvious way is based on the tomographic approach, where several diffraction patterns of the same sample are acquired at different orientations, giving a sufficient amount of 3D information in the reciprocal space to perform a 3D phase retrieval process via suitable algorithms (Miao *et al.*, 2006 ▸; Jiang *et al.*, 2010 ▸; Lundholm *et al.*, 2018 ▸; Loh *et al.*, 2010 ▸; Loh & Elser, 2009 ▸; Ekeberg *et al.*, 2015 ▸). In this respect, the recent advent of X-ray free-electron laser (XFEL) sources (Feldhaus *et al.*, 2005 ▸; Harmand *et al.*, 2013 ▸; Barty *et al.*, 2013 ▸; Chapman *et al.*, 2006 ▸) has opened new routes for characterizing objects that have thus far remained elusive. XFELs offer ultra-short and ultra-high-intensity pulses, enabling a meaningful scattering signal to be recorded before the object is destroyed, a scheme that has therefore been termed ‘diffraction before destruction’ (Chapman *et al.*, 2014 ▸). As a result, however, each sample can only provide a single diffraction pattern before being destroyed by the laser radiation. Thus, the 3D tomographic approach is viable only if many replicas of the same sample are available (Ekeberg *et al.*, 2015 ▸). Although additional shape information or symmetry constraints on the sample can in principle allow for shape retrieval from a single SAS diffraction image (Xu *et al.*, 2014 ▸), a full 3D reconstruction of non-replicable samples with unconstrained shapes is impossible to perform with SAS experiments. The requirement for additional constraints for reconstructing 3D information from a SAS experiment is a result of the fact that the magnitude of the maximum momentum transfer 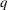 acquired by the scattering detector is much smaller than the radiation momentum 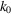 . Thus, as intuitively presented by Barke *et al.* (2015 ▸), the acquired momentum transfers lie essentially in the plane orthogonal to the beam propagation direction [see also Fig. 3(*b*) in Section 2], and the sample’s depth information is, in practice, completely lost.

The limitation to 2D-only information can be overcome in the wide-angle scattering regime (WAS), where the 2D diffraction patterns contain 3D information. In fact, because of the comparable magnitudes of the momentum transfer 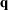 and the wavevector 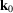 , the component of the momentum transfer parallel to the beam propagation direction, 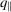 , which carries depth information, is non-negligible [see Fig. 3(*a*) in Section 2]. As shown by Barke *et al.* (2015 ▸), in this scenario different parts of the scattering pattern carry details about different 2D projections of the density – establishing the possibility of extracting partial tomographic information from a single image. The primary shortcoming of experiments in the WAS regime is that the scattering patterns cannot be converted into shape information in such a straightforward way as in the SAS regime, where the field represents the 2D FT of the density projection. Some attempts to numerically invert single WAS patterns have been made (Raines *et al.*, 2010 ▸): however, the stability and reliability of such approaches are still debated within the community (Wang *et al.*, 2011 ▸; Miao, Chen *et al.*, 2011 ▸).

Therefore, the forward-fitting approach, where a measured scattering pattern is compared with scattering simulations for appropriately parameterized sample shapes, is currently the most general and practicable approach to invert CDI data taken under WAS conditions. To perform such a forward-fitting analysis, a model that describes the sample’s morphology depending on a set of free parameters has to be selected. Then, those parameters’ values are varied using stochastic and/or deterministic optimization algorithms to minimize the discrepancy between the experimental diffraction data and the scattering simulation. In this procedure, the simulation of scattering patterns is the most challenging and computationally expensive task. In fact, optimization routines usually require thousands of optimization steps. For each step, the gradient of the optimization target has to be computed, and this involves a number of simulations that increases with the number of free parameters. Depending on the complexity of the sample’s model, this translates into a number of 10^4^ to 10^6^ simulations for the analysis of a single diffraction pattern, highlighting the urgent need for fast forward-simulation approaches.

If the simulation runtime is uncritical, *e.g.* for benchmarking purposes, or for cases with high symmetry, several approaches are available that enable computation of the exact solution to the scattering problem. The first method is based on the analytical solution for sufficiently simple geometries, such as the Mie solution to the Maxwell equations (Hahn, 2009 ▸), with which the scattered far-field can be calculated as a series expansion into vector wave harmonics up to arbitrary accuracy. However, such analytically motivated treatment is only applicable to simple sample shapes, like a sphere (Bohren & Huffman, 2008 ▸) or a coated sphere (Aden & Kerker, 1951 ▸). A second option is to compute the scattering by solving Maxwell’s equations numerically, *e.g.* via the finite-difference time-domain (FDTD) method (Taflove, 1980 ▸; Varin *et al.*, 2012 ▸) or using Green’s function based approaches such as the discrete dipole approximation (DDA) (Purcell & Pennypacker, 1973 ▸; Sander *et al.*, 2015 ▸). These numerical methods allow simulations of light–matter interaction with no restrictions on the sample’s shape. However, FDTD or DDA calculations of the scattered electric field are computationally cost intensive. The whole computational domain has to be represented on a grid at a sufficiently fine scale, and the temporal evolution of the field (FDTD case) or the iterative solution for the field’s evolution (DDA case) have to be calculated. The demanding computational conditions render the methods aiming at the unrestricted full solution of Maxwell’s equations impractical for the use case of simulating more than a few diffraction images. In particular, their time to solution is, even in the best cases, of the order of tens of seconds, such that a full imaging routine based on forward fitting would require from days to months to be completed for a single diffraction pattern. Therefore, suitable approximate methods are highly attractive for data analysis of wide-angle CDI. In this paper we present the Scatman, a fast, flexible and intuitive approximate simulation suite capable of providing scattering simulations three to five orders of magnitude faster than exact methods.

**Figure 1:**
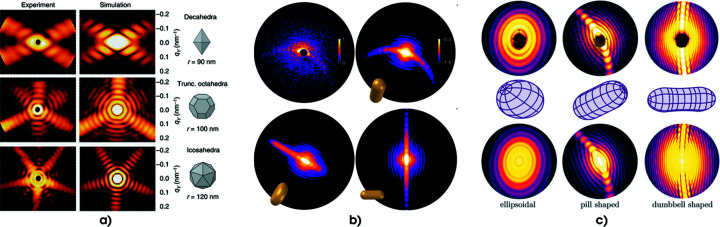
Examples extracted from previous work that made use of the Scatman approach for wide-angle scattering data analysis. In (*a*), adapted from Barke *et al.* (2015 ▸), soft X-rays are used to study the 3D structure of silver nanoparticles, by comparing the experimental data with the simulation. In (*b*), adapted from Rupp *et al.* (2017 ▸), comparisons between experimental data and simulations demonstrated the feasibility of coherent diffraction imaging with high harmonic generation sources. In (*c*), adapted from Langbehn *et al.* (2018 ▸), a fitting between the Scatman result and experimental diffraction patterns revealed the 3D shapes of superfluid helium nanodroplets.

The Scatman’s core was originally conceived by Barke *et al.* (2015 ▸) and computes wide-angle coherent scattering images of isolated samples. It has already proven successful for data analysis of WAS experiments (Barke *et al.*, 2015 ▸; Rupp *et al.*, 2017 ▸; Langbehn *et al.*, 2018 ▸; Zimmermann *et al.*, 2019 ▸): examples ranging from silver nanocrystals to spinning superfluid helium droplets are depicted in Fig. 1 ▸. However, a formal presentation of the approach and a comprehensive evaluation of its performance have never been disclosed. This paper aims to fill this gap, by presenting the Scatman approach in a refined, generalized and concise form, accompanied by the public release of its software implementation, *PyScatman*.

The two following sections of this paper are dedicated to the analytical framework and motivation of the approach, based on the multi-slice Fourier transform (MSFT) technique (Cowley & Moodie, 1957 ▸; Self *et al.*, 1983 ▸; Reinhard *et al.*, 1997 ▸; Hare & Morrison, 1994 ▸; Barke *et al.*, 2015 ▸), and its translation into a numerical form. Section 4 focuses on the comparison between the simulation results of the Scatman and exact, analytical calculations based on Mie theory for a spherical sample. It provides insight into the region of applicability of the Scatman, whose results can be quantitatively close to the exact solution or just qualitatively usable, depending on the sample’s properties. The final sections, Sections 5 and 6, present our Scatman reference implementation, called *PyScatman*, published along with this paper as open-source software. *PyScatman* is released as a Python module that provides an easy interface to the user and incorporates state-of-the-art programming techniques to yield a high computational efficiency.

## 2. The Scatman routine

**Figure 2:**
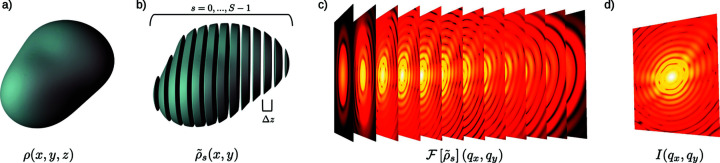
Schematic representation of the Scatman’s MSFT approach. In (*a*), the sample as a whole is defined by its scattering strength ρ, which depends on the spatial distribution of the complex refractive index *n*. In (*b*), the sample is split into *S* slices, where, for each slice *s*, the scattering density 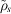 is determined by the slice’s optical properties. In (*c*), the scattered far field is computed for each slice *s*. In the last step (*d*), the scattering of the slices is summed with a phase correction and subsequently squared to simulate the recorded diffraction pattern on the detector. For clarity of presentation, (*c*) only shows the scattering signal’s squared amplitude for every slice, while the actual scattered wavefield is still a complex function at this point.

The Scatman is based on the MSFT approach, originally developed for electron scattering (Cowley & Moodie, 1957 ▸; Self *et al.*, 1983 ▸; Reinhard *et al.*, 1997 ▸). The MSFT routine has already been applied in X-ray diffraction experiments for fixed targets (Hare & Morrison, 1994 ▸), as well as for recovering the topology of individual silver and helium nanoparticles in free flight (Barke *et al.*, 2015 ▸; Langbehn *et al.*, 2018 ▸). A schematic overview of the MSFT method is shown in Fig. 2 ▸. Roughly speaking, the simulation is based on the partitioning of the spatial domain into slices [Figs. 2 ▸(*a*) and 2 ▸(*b*)]. The scattering contribution from each slice is computed independently via an FT operation [Fig. 2 ▸(*c*)] and then summed with an appropriate phase correction to compose the final scattering pattern [Fig. 2 ▸(*d*)]. This section briefly revisits the mathematical derivation of the approach, particularly focusing on how the effects of the sample’s refractive index are effectively incorporated into the Scatman’s simulation.

**Figure 3:**
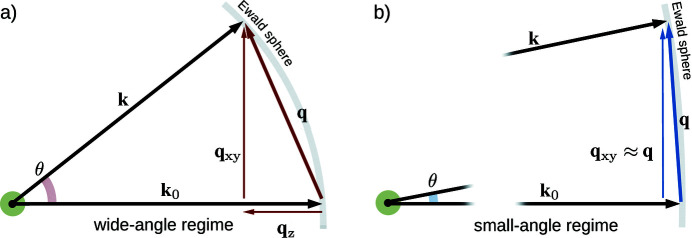
Schematic view of wide-angle and small-angle regimes and of the notation used in this paper for the momentum vectors. The momentum transfer 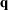 is defined as the difference between the incoming wavevector 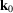 and the scattering vector 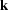 . 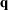 can be decomposed into its axial component 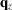 , *i.e.* the component parallel to the incoming radiation assumed to travel along the *z* axis, and 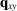 . The wide-angle regime is depicted in (*a*), where the axial component 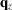 is non-negligible thanks to the large scattering angle θ. For comparison, (*b*) shows the same scheme in the small-angle regime, where the scattering angle θ is sufficiently small to neglect the axial component of 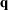 . In both (*a*) and (*b*) the Ewald sphere is shown in gray.

For deriving the method, we start from the well known Born approximation (Born, 1926 ▸), which defines the scattered field 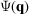 in the far-field condition as

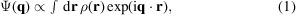

where 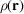 defines the scattering strength in space and 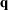 is the momentum transfer, schematically shown in Fig. 3 ▸. The integral in equation (1), which is in practice a 3D FT of the scattering strength 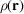 , can be rewritten in the following form: 

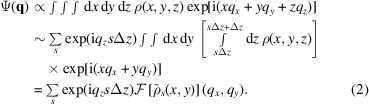

The first step in equation (2) is the explicit formulation of equation (1) in Cartesian coordinates, where the coordinate system is chosen such that the *z* axis is parallel to the beam propagation direction; from now on, this axis will also be referred to as the axial direction. The second step of the equation is the approximation of the integral along **z** with a discrete summation over the slices with integer index *s*; this approximation holds given the condition that 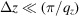 . As depicted in Fig. 2 ▸(*b*), this operation represents a partitioning of the spatial domain into slices of size 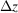 along the axial direction, this being the core of the MSFT approach. In the last step, the integral over the *x* and *y* directions is rewritten as a 2D FT. Moreover, the integral of the scattering strength ρ over the slice *s* in the axial direction is defined as 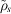 .

As long as only monochromatic radiation with momentum 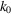 is considered and the scattering event is assumed to be completely elastic, it is convenient to rewrite the axial component of the momentum transfer 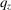 as a function of 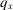 and 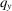 : 

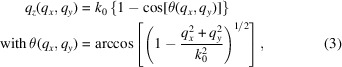

where θ is the scattering angle. Equation (3), which can be intuitively derived from Fig. 3 ▸(*a*) through geometrical considerations, enables equation (2) to be rewritten as 

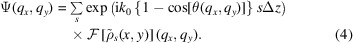

The scattered field 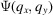 in equation (4) is the sum of the scattering contributions from all *s* slices, with a phase factor that depends on the scattering angle and on the slice’s position on the *z* axis.

We continue the derivation of the method by defining the scattering strength 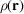 . In particular, at high photon energies, the strength of the scattering is related to the number of electronic charges that contribute to the scattering. These are described by the dielectric polarization density 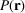 , defined as

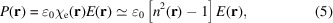

where 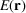 is the electric field and 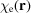 is the electric susceptibility. The latter has been rewritten as a function of the complex refractive index *n*, exploiting its relationship with the relative permittivity 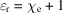 , which is equivalent to the squared refractive index for non-magnetic materials, *i.e.* 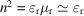 . In this way, any magnetic effect is neglected, for example in the interaction of magnetic materials with polarized light. Still, light polarization plays a role also for dielectric materials. However, the effects of linearly polarized light become relevant only for scattering angles above 30° (Bohren & Huffman, 2008 ▸), and thus above the usual range of wide-angle CDI experiments (Barke *et al.*, 2015 ▸; Langbehn *et al.*, 2018 ▸). The current version of the Scatman assumes non-polarized radiation, but its inclusion in the routine is under study.

The Born approximation in equation (1) assumes that the incoming electric field is not affected by the presence of the sample, and considers a planar wave with constant amplitude, momentum and phase along the full path. In this view, the scattering strength defined in equation (1) is proportional to the electric susceptibility, *i.e.* 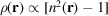 , allowing the definition of the scattering strength for a slice in equation (4) as 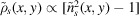 , where 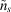 are the optical properties averaged over the slice thickness. However, especially when considering WAS, the refractive index of the sample will modify the field. It is possible to partially take into account this effect by defining the scattering strength for a given slice *s* in the following form: 

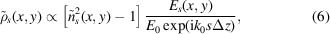

where 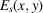 is the field actually impinging on slice *s* of the sample, while 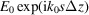 is the field as it would travel unaffected by the presence of the sample [the field taken into account by the Born approximation in equation (1)]. The ratio between the two fields can be interpreted as a correction applied to the unmodified field assumed in the first Born approximation, making 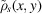 in equation (6) an effective scattering strength. This correction factor allows us to approximately include the effects of the sample’s optical properties on the electric field incoming to the slice, while the scattered field is still the unaffected one considered in the Born approximation.

For a more intuitive presentation of how the incoming field 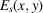 impinging on the slice *s* is treated in the Scatman approach, it is now convenient to rewrite the sample’s refractive index *n* in the following form: 

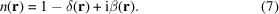

Here, δ defines the deviation of the real part of *n* from unity, and is responsible for the change of the light’s phase velocity in the sample, causing also the phenomena of refraction and reflection. On the other side, β, often called the extinction or absorption coefficient, defines how much the radiation is damped when traveling in the sample (Lambert, 1760 ▸; Beer, 1852 ▸). This notation for the refractive index is convenient in the X-ray regime, where 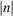 is very close to unity, and will be extensively used in this paper.

An exact description of how the field distribution in the sample is affected by δ and β, regarding the field’s amplitude, phase and propagation direction, is highly demanding and essentially requires again the full solution of the scattering problem. However, in the limit of sufficiently small δ and β, it is possible to assume the ‘projection approximation’ (Paganin, 2006 ▸), reducing the expression for the propagation of the electric field 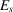 at slice *s* to the following form: 

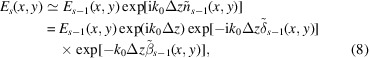

where 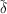 and 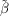 are the values of δ and β averaged over the slice thickness 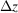 , and 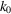 is the radiation wavenumber. This approximation locally assumes an axial propagation through a homogeneous medium. Equation (8) recursively describes how the field impinging on slice *s* is modified by taking into account the effects of all the preceding slices. A first strong assumption made by equation (8) is that the optical properties must vary slowly. In particular, the variation of δ has to be sufficiently small that one can neglect changes in the field propagation direction due to refraction and reflection, *i.e.* the electric field always propagates in the axial direction, even within the sample. Moreover, δ and β are assumed to be sufficiently small that one can neglect their influence on the radiation scattered by the preceding slices, *i.e.* secondary scattering is completely neglected (for the discussion of the resulting limitations, see Section 4). In practice, at a given slice *s*, 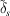 introduces a phase shift in the field, while 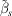 exponentially damps the field magnitude.

Finally, the scattering strength [equation (6)] has to be inserted into equation (4). The electric field in the denominator of equation (6), which is independent of 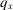 and 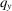 , can be pulled out of the FT simplifying the global phase pre-factor of the slice. This operation, combined with the formula for the approximated field propagation in equation (8), yields the main equation of the Scatman approach: 

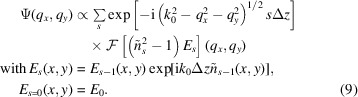

Here the definition of the scattered field is based on the spatial distribution of the sample’s optical properties. Although equation (9) predicts the scattered electric field, only its squared amplitude 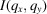 = 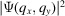 is physically measured in CDI experiments (Gaffney & Chapman, 2007 ▸; Chapman & Nugent, 2010 ▸; Seibert *et al.*, 2011 ▸; Ekeberg *et al.*, 2015 ▸) and should be taken into account for actual simulations.

## 3. Numerical implementation

**Figure 4:**
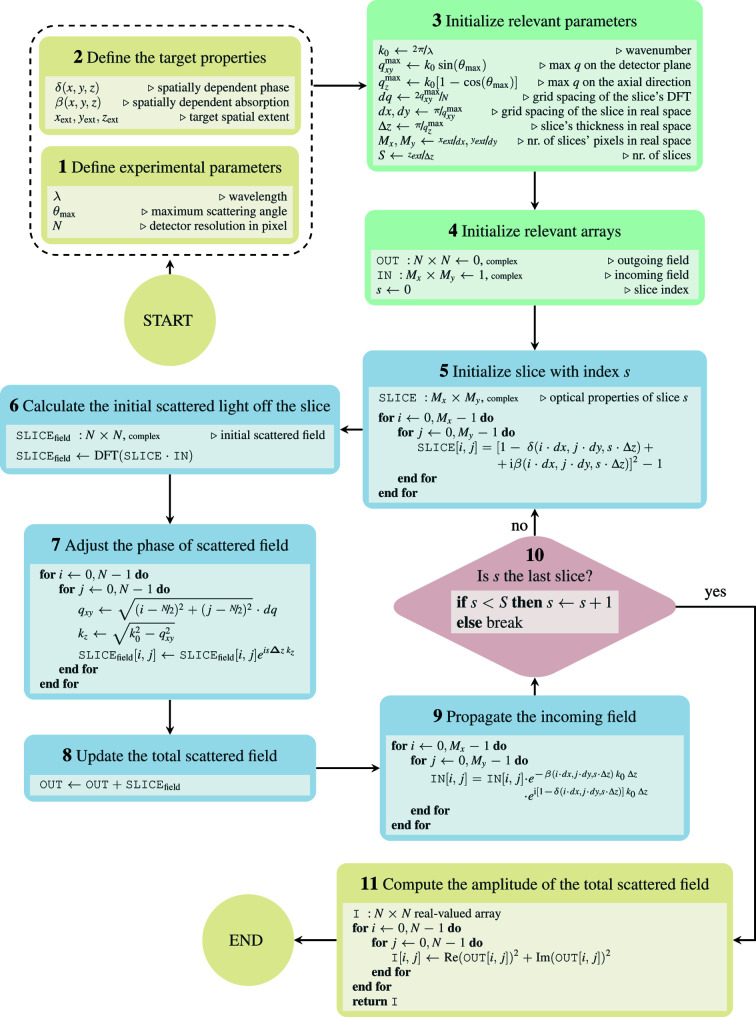
A flowchart of the conceptual core of the Scatman in numerical form is shown. Yellow blocks indicate I/O operations. Green blocks contain data preparation. The main loop of the program, which carries out the majority of calculations, is highlighted in blue. Each block contains a pseudo-code schematically showing the numerical calculation. The abbreviation DFT stands for discrete Fourier transform, practically computed through the fast Fourier transform algorithm (Cooley & Tukey, 1965 ▸).

In this section the concrete numerical implementation of the Scatman is provided. A flowchart of the program is shown in Fig. 4 ▸, and its blocks are described in the following paragraphs.

### 3.1. Setting up the virtual experiment

*Read-in the user-defined parameters (Blocks 1 + 2)*. At the beginning of the program, the user-defined input parameters are read in. These include experimental details (Block 1) like the irradiation wavelength λ, the maximal scattering angle 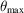 and the detector resolution *N* in pixels. *N* = 1000 will result in a virtual detector of size 1000 × 1000 pixels. The virtual detector is centered on-axis in the **z** direction, and every virtual pixel has the same angular cross section. Furthermore, the target is defined via a spatially dependent refractive index (δ and β) and the concrete dimensions of the target ( 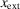 , 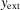 , 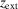 ), as listed in Block 2. With this set of parameters the experimental setup is uniquely defined.

*Initialization (Blocks 3 + 4)*. Before entering the program’s main loop, additional parameters are derived from the user-defined parameters, and the relevant arrays are initialized.

Here, the maximal components of the scattering vector on the *xy* plane (also called the detector plane) and in the axial direction, 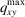 and 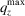 , are calculated. As the FT in equation (9) is numerically computed in the discrete form, 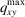 is necessary to assign a corresponding size d*x* and d*y* to the slices’ pixels in real space, and consequently its spatial extension in pixels, 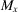 and 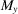 . The same applies to the axial direction, where 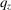 defines the slice thickness 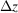 , and thus the total number of slices *S*.

Two complex numerical arrays are then initialized to hold the 2D wavefronts of the outgoing and the incoming wavefields (Block 4). While the outgoing wavefield is initialized to zero, the incoming field is initialized to 1, which is equivalent to a plane wavefront. The assumption of a perfectly coherent incoming beam, in both space and time, is strictly enforced from the mathematical foundations of the Scatman approach. However, variations in amplitude and phase are, in principle, not forbidden, and their inclusion in future releases of *PyScatman* are currently under study. The desired diffraction image can now be iteratively computed within the program’s main loop.

### 3.2. The main loop

The main loop is the core of the program. Every iteration within it calculates the scattering contribution of one slice of the sample. Each slice’s input field is the original plane wave shaped by the optical properties of the sample up to the slice of interest. The scattered radiation is then corrected with a proper phase factor.

*Calculating the local scattered field (Blocks 5 + 6 + 7)*. The first step required to compute the slice’s scattered field is to render the slice’s scattering potential (Block 5) through the computation of the sample’s optical properties at the proper spatial coordinates. Calculating the slice’s scattering contribution is, then, the subject of Blocks 6 and 7, where both multiplicative terms are treated in their own blocks. First, the FT of the product between the incoming field and the slice yields the far-field scattering contribution of the latter (Block 6). Then, the proper phase correction is applied (Block 7). Note that the scattering vector’s components are derived in pixel units from their off-axis distance relative to the *z* axis. The final wavefield is then stored in the complex array SLICE_f__ield_ .

*Updating the total scattered field and computing the incident field for the next slice (Blocks 8 + 9)*. The total scattered field is updated in Block 8 by adding the scattering contribution of slice *s* to those of all the previous slices.

Then, in Block 9, the incoming field for the next slice is prepared by propagating the field through slice *s* along the *z* axis, following the definition in equation (8). The decoupling of the total scattered field (Block 8) and the incoming field for any subsequent slice (Block 9) enforces a central assumption within the Scatman: multiple sequential scattering events are not allowed to occur.

### 3.3. Preparing the output of the Scatman

After the main loop iterates over all slices in the virtual medium, *i.e.* when Block 10 reaches the loop break condition, the Scatman’s final piece of code prepares the output to match the experimental conditions. In the simplest case, this is just computing the absolute squared value of all slices’ total scattered field (Block 11). However, it can include the modeling of detector artifacts, stray light during the experiment or any other experimental effect that may affect a real scattering pattern.

## 4. Evaluation of the Scatman using exact simulations

As highlighted in Section 1, the Scatman program is an alternative to the computationally intensive, but versatile, numerical simulations such as FDTD or DDA methods, and to Mie’s fast, but topologically restrictive, analytical solutions to Maxwell’s equations. However, as underlined in Section 2, there is a trade-off between the Scatman’s capability of being both fast and versatile and the accuracy of the simulation results, which depends heavily on the choice of the simulation parameters.

During the mathematical formulation of the approach in Section 2, some approximations were involved. Most of them imply the assumption that the optical properties of the sample of interest only differ slightly from those of the surrounding medium (assumed here to be a vacuum). Therefore, the Scatman approach is unable to quantitatively reproduce features of the scattering images when relatively large variations of the refractive index are present, as usually happens, for example, close to electronic resonances or for materials with a high scattering cross section. Still, probing how small the variation of the refractive index has to be is of great interest to the user, to decide whether it is preferable to rely on alternative and more accurate methods for data analysis. This section provides an overview of the capabilities and limitations of the Scatman program, where we compare the simulation results with analytical diffraction patterns obtained via Mie theory. The comparison is made by considering a spherical sample to disentangle as much as possible the effects of the optical properties from those produced by morphological features. For an overview on how the method used for the Scatman compares with exact methods in the case of complex sample architectures, readers are referred to Barke *et al.* (2015 ▸) and Langbehn *et al.* (2018 ▸).

**Figure 5:**
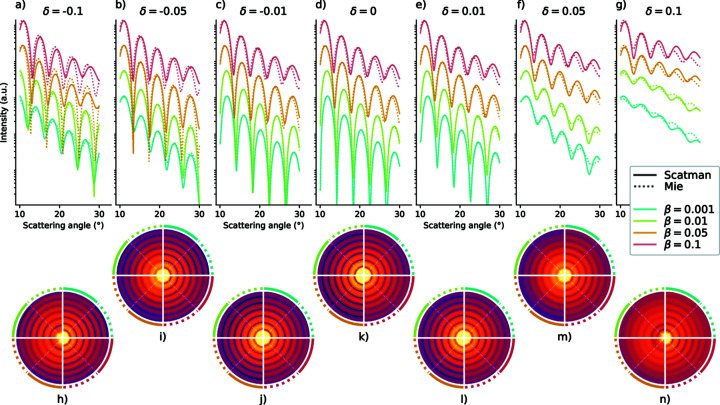
Comparison of radial profiles between the Scatman and Mie calculations. The figure is split into two rows that share a common legend, which is placed in between the rows. In the upper part, from (*a*) to (*g*), 28 radial profiles are shown that correspond to 28 combinations of δ and β for a fixed spherical target of radius 7λ. For each combination of δ and β the Scatman approximation along with the exact Mie results is plotted in solid and dotted lines, respectively. In the lower part, the seven subplots from the top part are translated into seven diffraction images in (*h*) to (*n*), where the intensity of the scattering signal is encoded in logarithmic color scale. Every diffraction image is partitioned into eight segments. These eight segments correspond to the eight line plots shown in the associated plot from the top row. Every δ/β pair combination takes up a quarter of every diffraction image, where the solid and dotted lines surrounding the diffraction image indicate that the quarter is showing either the approximation of the Scatman program (solid line) or the exact Mie solution (dotted line).

Fig. 5 ▸ shows the results for 28 scattering simulations for a spherical target, with a different pair of δ and β values each. The figure is split into two rows that show the radial profiles and the diffraction patterns. Both rows share a common legend, which is placed in between: solid lines represent the Scatman result, dotted lines the Mie solution, and the colors indicate different β values. The top row shows seven subplots (*a*) to (*g*), where each subplot shows the scattering angle dependence of the scattered light from a spherical particle for a fixed value of δ and four values of β. The choice to limit the scattering angle to a range between 10 and 30° corresponds to typical experimental scenarios for CDI experiments within the WAS regime (Rupp *et al.*, 2017 ▸; Langbehn *et al.*, 2018 ▸; Barke *et al.*, 2015 ▸). In every subplot and for every δ and β pair, two calculations are shown: the solid line is the approximation of the Scatman program and the dotted line is the exact Mie solution. The radius of the spherical target used for the simulations is fixed at 7λ, which enables one to see the signature of different optical properties, and to distinguish the maxima and minima of the interference as well. For a fair comparison between the two simulation methods, a normalization factor has to be defined: in this case, both Mie and Scatman profiles were normalized on their integral value computed between 10 and 30°.

In the particular case of a spherical target, assuming non-polarized light, the simulated diffraction image is identical in all scattering directions. This symmetry property is exploited in the bottom row of Fig. 5 ▸. The seven subplots from the top row are translated into seven diffraction images (*h*) to (*n*), where every diffraction image is partitioned into eight segments. These eight segments correspond to the eight line plots provided in the associated subplot from the top row. Every δ/β pair combination takes up a quarter of every diffraction image, where the solid and dotted lines surrounding the diffraction image correspond to the approximation of the Scatman program (solid line) and the exact Mie solution (dotted line).

Therefore, the bottom row does not add new data to the figure but provides insight into the appearance of the scattering image. Furthermore, it enables a qualitative assertion on the diffraction images, which is often sufficient to deduce the sample’s underlying topological properties. (Rupp *et al.*, 2017 ▸; Langbehn *et al.*, 2018 ▸; Barke *et al.*, 2015 ▸).

The simplest case during this evaluation is for δ = 0 in combination with the smallest β value (0.001). There, the wavefield that propagates throughout the medium is identical in phase to a reference field propagating through the surrounding vacuum and only very weakly absorbed. The corresponding Scatman and Mie calculations are shown in blue in Figs. 5 ▸(*d*) and 5 ▸(*k*). The solid and dotted blue indicated slices in the diffraction image in (*k*) are indistinguishable by eye, just as the radial profiles in (*d*) are. However, when increasing the absorption from 0.001 to 0.01, slight deviations become visible at high scattering angles, where the Scatman program produces a radial profile in which the maxima are shifted towards higher scattering angles, and the amplitude is slightly too high compared with the analytical results.

This behavior is core to all Scatman approximations where the absolute value of δ is comparably small ( 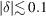 ). With increasing absorption, the Scatman overestimates the signal’s total amplitude and shifts the extrema at larger scattering angles towards even larger scattering angles. Therefore, when δ is comparatively small, the quality of the Scatman’s simulation is anticorrelated with the absorption in the medium.

The scenario strongly varies when larger values of δ are considered. There, the Scatman’s behavior is more complicated, mostly due to the appearance of intricate resonance effects that arise from the interplay between the target’s geometry and the wavefield. Such resonance effects are more pronounced for positive values of δ (refractive index smaller than unity), for example, observed in the atomic near-resonance regime, where the photon energy dependence of δ resembles a Fano profile. Thus, the assertion concerning the δ dependence must be split for positive and negative values. At negative values, broadly speaking in the off-resonance case, the deviations between the Scatman and the Mie simulation are mainly due to an overestimation of the amplitude with a relatively tiny shift of the extrema positions in the radial profiles. However, at positive values of δ, the deviations between the two simulations are significant. With δ values above 0.1, not shown here, the resulting radial profiles differ wildly from one another.

Therefore, besides the anticorrelation with β for small δ, the second deduction that can be made here is that the Scatman produces worsening diffraction images with a more positive δ (refractive index smaller than unity). For the specific case presented here, the pivotal point for this to happen is roughly for 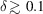 .

So far, the comparison between the Scatman’s results and Mie theory has been restricted to a fixed target size. However, the features of scattering images of isolated nanoparticles vary significantly, depending also on the targets’ size (Mie, 1908 ▸; Bohren & Huffman, 2008 ▸; Rupp *et al.*, 2014 ▸). Thus, a more exhaustive comparison, which also includes size effects, is presented in the supporting information.

Concluding, the approximation employed by the Scatman program produces in most cases diffraction patterns of very high quality compared with the analytical Mie solution for spherical particles. In general, the quality of the routine is best when the phase term in the refractive index is small ( 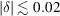 ). Then, only minor deviations are observed and the Scatman’s approximation could even be used as a replacement for the Mie theory based solution. With increasing absolute values of δ, the quality deteriorates as well, where larger positive values of δ yield worse results than larger negative values. At low δ values, the absorption (β) is anticorrelated with the quality of the approximation, yielding high-quality diffraction images when absorption is low. This relationship, however, is reversed for larger absolute values of δ, where a larger amount of absorption yields a better comparison with the Mie theory calculations.

The exact range of optical properties for which the Scatman approach is usable is highly dependent on the scope of the simulation and its application. For this reason, we encourage users to take the values given in this section as purely indicative, and to directly check the applicability in their specific use cases.

The next section introduces *PyScatman*, a high-level Python front-end for the Scatman method.

## 5. *PyScatman*: a high-level Python front-end

In this section we present and explain the reference implementation of the Scatman in the form of a Python module, called *PyScatman*. The source code is available under the MIT license (https://spdx.org/licenses/MIT.html) at https://gitlab.ethz.ch/nux/numerical-physics/pyscatman, while the documentation can be found at https://nux-group.gitlab.io/pyscatman/.

The module is written in C++ (The C++ Standards Committee, 2017 ▸) with bindings in Python using the *PyBind11* C++ library (Jakob *et al.*, 2019 ▸). This hybrid approach enables us to maintain the highest possible simulation speed via compiled C++ code while keeping a Python-only user-friendly interface. The implementation is highly parallelized for multi-core CPUs, and takes advantage of Nvidia GPU accelerators via the CUDA library (Nickolls *et al.*, 2008 ▸). In the current version, *PyScatman* performs all the computations in single floating point precision (32 bit).

In Section 5.1 a fundamental example is provided and explained. There, an experiment is set up, an ideal detector is defined and a simple shape is generated.

Building on this, Section 5.2 provides a more advanced example, where three shapes are generated using three different methods, and where a detector that simulates photon statistics is used. This second example is meant to highlight the great flexibility offered by the *PyScatman* module in terms of defining a target’s shape.

Finally, in Section 6, the implementation is extensively benchmarked with respect to its execution time on either the CPU or the GPU using various shapes.

### 5.1. A fundamental example

**Figure 6:**
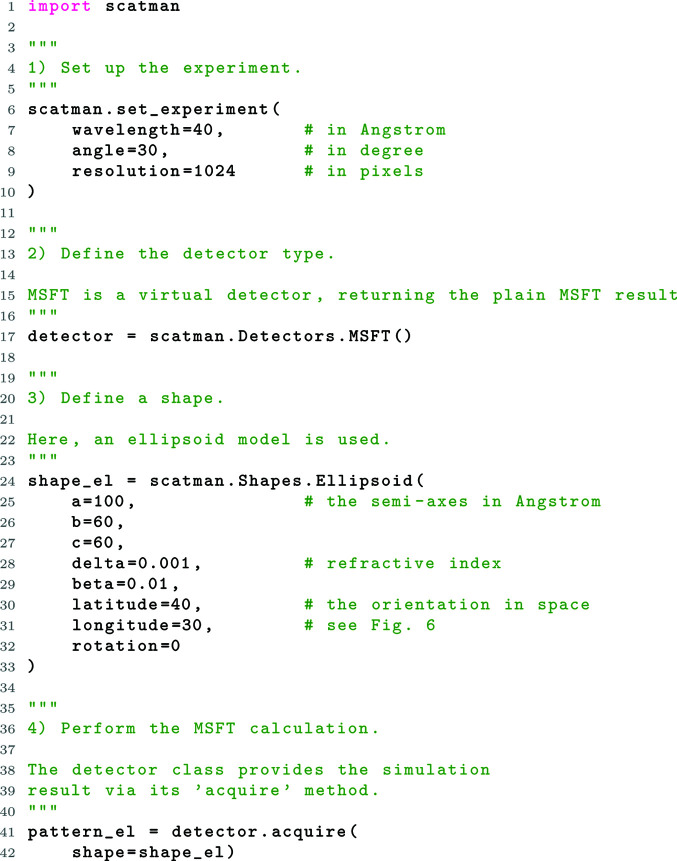
Listing 1: a fundamental *PyScatman* example. Here, we set up the experiment, define a detector function and calculate the MSFT simulation for an ellipsoidal sample.

In this section, a fundamental example is provided and explained. We demonstrate the basic functionality and show the easiest way to define the target’s shape (see Fig. 6 ▸).

For discussing the elements in the script we will refer to the line numbers.

*Define an experiment (lines 1 to 10)*. After the Scatman module is imported, the experimental conditions are set up by defining the irradiation wavelength in ångstöms, the maximal scattering angle in degrees and the desired detector resolution in pixels (px). Within *PyScatman*, there is no preferred length unit: the only requirement is to keep the same unit (Å in this example) throughout the whole script. An additional optional parameter that defines the radiation intensity is described later in the advanced example in Section 5.2.

*Define a detector (line 17)*. The Scatman module provides three detector types: MSFT, Ideal and MCP. MSFT is a virtual detector, which directly yields the plain MSFT calculation, while the Ideal one attempts to model realistic photon statistics and noise augmentation. The Ideal detector is described as part of the advanced example in Section 5.2. Finally, *PyScatman* provides the MCP class, which aims to simulate a scattering detector based on a microchannel plate (MCP) (Wiza, 1979 ▸), often used in CDI experiments (Bostedt *et al.*, 2010 ▸; Rupp *et al.*, 2017 ▸, 2020 ▸; Langbehn *et al.*, 2018 ▸). The MCP class is not described here as it is beyond the scope of this paper. However, a full description can be found in the *Detectors* section in the official *PyScatman* documentation (https://nux-group.gitlab.io/pyscatman/detectors.html).

In this fundamental example, the MSFT detector is used, which returns the exact MSFT calculation.

**Figure 7:**
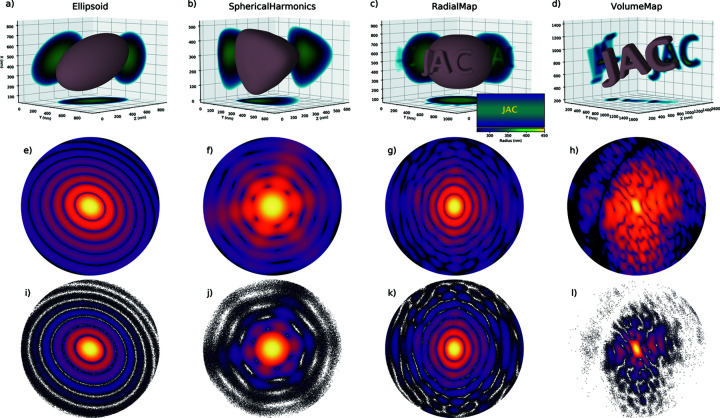
Rendering of the shapes and their respective simulated diffraction patterns using two different detectors. From (*a*) to (*d*), the 3D rendering of the shape objects, defined using the Ellipsoid (*a*), SphericalHarmonics (*b*), RadialMap (*c*) and VolumeMap (*d*) models. The RadialMap example in (*c*) has an inset showing the array that was used for creating the shape, where the radius information is color coded. The corresponding diffraction patterns of samples (*a*)–(*d*), computed by *PyScatman* via the MSFT detector, are shown in (*e*)–(*h*). The third row, from (*i*) to (*l*), shows instead the equivalent simulation results provided by the Ideal detector. Here, the effects of photon statistics are clearly visible, along with the dependence on the value of the absorption coefficient. For example, the samples in (*b*) and (*c*) have an absorption coefficient 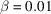 and 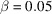 , respectively, which reflect a signal-to-noise ratio higher in (*k*) than in (*j*). Refer to the examples in the main text for further details.

*Define a shape (lines 24 to 33)*. *PyScatman* comes with several pre-defined sample shape models, each with specific parameters that define their appearance.^1^ The sample described in listing 1 (Fig. 6 ▸) is of ellipsoid shape and is shown in Fig. 7 ▸(*a*). Note that the three axes of the ellipsoid are given in units of Å, as they must be consistent with the definition of the radiation wavelength set at line 7.

**Figure 8:**
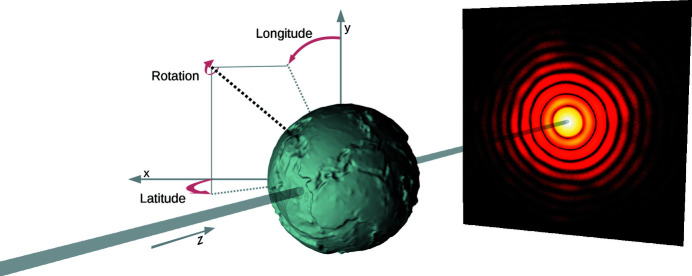
Schematic of the orientation in space for the latitude, longitude and rotation properties of the shapes in the *PyScatman* module. A shape model is oriented by setting the direction in space of its main axis. This direction is defined through latitude and longitude parameters, expressed on a reference system where 90° latitude indicates a direction towards the incoming beam and, thus, −90° latitude is towards the detector. The equator of the reference system, at 0° latitude, lies on the plane orthogonal to the beam. Once the shape’s main axis is oriented, a rotation is applied to the sample along this axis. Here, the simulated sample reflects Earth’s elevation, to highlight the large flexibility that the *PyScatman* module offers in terms of how to define the target shape. The sample was modeled though an adaptation of *ETOPO1* data (Amante & Eakins, 2009 ▸), here in an exaggerated scale, which provides Earth’s elevation as a function of the Earth’s coordinates, and simulated by the use of the RadialMap shape model. See Fig. 9 ▸ for further details on how to define such a shape.

All shapes have a delta, beta, latitude, longitude and rotation preference, which define their refractive index inside the sample and their orientation in space (see Fig. 8 ▸ for a schematic on how the coordinates are defined). There, the latitude and longitude properties follow the standard convention also used for defining the coordinates on Earth, where the north–south axis is along the *z* direction (solid gray line).

*Calculate the MSFT (lines 41 to 42)*. After having defined a shape and a detector for an experiment, we can use the acquire method of the detector class to calculate the MSFT-based diffraction image. In this example, the variable pattern_el is a *Numpy* array with dimensions 1024 × 1024, as this was the resolution set at line 9. The final calculation of the diffraction image is shown in Fig. 7 ▸(*e*).

### 5.2. A more advanced example

**Figure 9:**
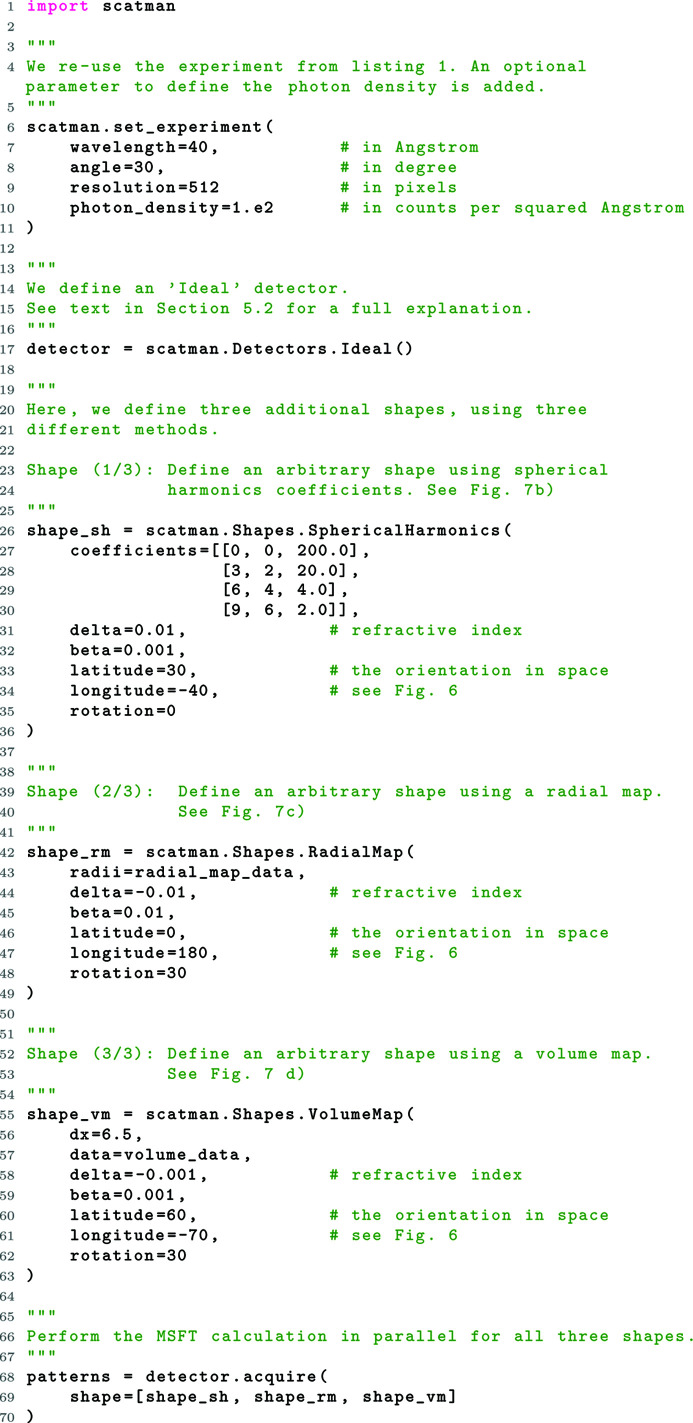
Listing 2: a more advanced *PyScatman* example. Here, we calculate the MSFT diffraction images for various samples whose shapes are each defined in a different way.

One of the main advantages of the *PyScatman* module is the flexibility with which any arbitrary shape can be defined. In addition to the pre-defined shapes introduced in Section 5.1, here we present three additional methods that *PyScatman* provides for defining an arbitrary shape: (i) Spherical­Harmonics, (ii) RadialMap and (iii) VolumeMap. All three methods are described in listing 2 (Fig. 9 ▸). *PyScatman* provides additional methods to define the sample’s shape [including the architectures presented by Barke *et al.* (2015 ▸) and Langbehn *et al.* (2018 ▸)] which are not discussed here, and more of them will be added in future releases.

*Defining an Ideal detector (lines 1 to 17)*. We import the *PyScatman* module and set up the same experiment as in listing 1 (Fig. 6 ▸), with the addition of the optional parameter photon_density which plays a role in the later-defined Ideal detector. The idea behind the implementation of the Ideal detector is that even a perfect real-life detector is subjected to Poisson statistics of photons, which augments the recorded diffraction images. In order to model this effect, an estimate of the number of scattered photons has to be calculated and then used to add the appropriate Poisson noise to the simulated diffraction pattern. A description of how this data augmentation is implemented in *PyScatman* is given in the supporting information.

*Shape (1/3) via spherical harmonic coefficients (lines 26 to 36)*. Any shape that is described by a radius as a function of the azimuthal and polar angles can also be defined using spherical harmonic coefficients. A notable example is the equipotential surface of the gravity potential of the Earth, which is termed the geoid and is defined using spherical harmonics (Barthelmes, 2009 ▸).

In general, the convention we use for the Laplace spherical harmonics ( 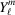 ) is defined as

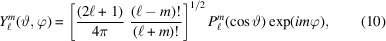

where *m* and ℓ are the order and degree of the harmonics, ϑ and φ are the azimuthal and polar angles within the spherical coordinate system, and 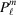 are the associated Legendre polynomials, defined as

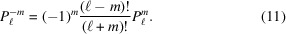


*PyScatman*’s SphericalHarmonics class expects a list of triplets, where the first value corresponds to degree ℓ, the second value to the order *m* and the third value to a scaling parameter with which 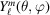 is multiplied. The final shape is then the sum of all triplets within the passed list.

The shape defined at lines 26 to 36 in listing 2 can be seen in Fig. 7 ▸(*b*), along with the calculated MSFT diffraction image for this shape in Fig. 7 ▸(*f*).

*Shape (2/3) via a radial map (lines 42 to 49)*. A second method for defining an arbitrary shape within *PyScatman* is to provide a 2D array of any size that holds the length of the radii for all values of both angles θ and φ, which can be interpreted as the latitude and longitude coordinates. For example, when an array with size 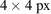 is passed, then these values define the radii of the shape at the θ values 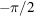 , 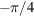 , 0 and 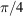 , and for φ at the values 0, 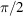 , π, and 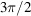 . These values are then linearly interpolated when the sample shape is rendered at the proper resolution, depending on the sample size and the experimental conditions defined at lines 6–11.

The shape of type RadialMap presented in the example, named shape_rm, is produced by the 2D radial map radial_map_data (of size 1920 × 960) given as an argument in line 43. The rendered shape can be found in Fig. 7 ▸(*c*), where an inset shows the used radial map. The MSFT calculation for this shape is shown in Fig. 7 ▸(*g*).

*Shape (3/3) via a 3D volume map (lines 55 to 63)*. The third method for defining an arbitrary shape is via a volume map. The VolumeMap class of *PyScatman* requires a 3D array of Boolean type (volume_data at line 59, here of size 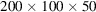 ), which can have any size. The dx parameter, then, defines the linear size of a single volume unit of the 3D array volume_data and, as usual, must be expressed in the same length unit as the wavelength. For example, if a 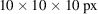 array with every value set as Boolean true is passed as data argument, and the dx argument is set to 2, we end up with a cubic shape of size 20 × 20 × 20 Å. If we want to scale up that cube by a factor of 2, we can set the dx property to 4, which results in a cube with doubled dimensions. The 3D rendering of the shape defined in this example is presented in Fig. 7 ▸(*d*), along with its MSFT simulation in Fig. 7 ▸(*h*). *PyScatman* also provides the possibility to perform simulations of non-uniform samples via a completely arbitrary voxel representation of the refractive index. This feature is not presented here for the sake of simplicity; readers may consult the software documentation for more information.

*Obtaining the results (lines 68 to 70)*. Finally, the simulation is performed for all three shapes. All shapes can be simulated through a single call to the Ideal detector’s acquire method, passing them as a list. This possibility is implemented for allowing the *PyScatman* module to better exploit parallel computing hardware (and especially multiple GPUs) when large data sets have to be simulated (see Section 6 for further details). The patterns array yields the simulations, formatted as a list of 2D arrays that contain the simulation result for the shapes shape_sh, shape_rm and shape_vm. These patterns are depicted in Figs. 7 ▸(*j*), 7 ▸(*k*) and 7 ▸(*l*), respectively, where the effects of photon statistics simulated by the Ideal detector are clearly visible.

## 6. Performance considerations

Our primary intention for the *PyScatman* module is to enable data analysis on diffraction patterns by forward-fitting the MSFT simulation with the experimental results, since classical Fourier reconstruction via phase retrieval methods is not possible for WAS. The model fitting approach consists of guessing a target’s shape, simulating its diffraction pattern, comparing it with the desired experimental data and then iteratively improving the guess until the MSFT simulation is sufficiently close to the experimental image. Such an optimization scheme is computationally expensive in its own right. Therefore, it is of the utmost importance to speed up the MSFT simulation as much as possible.

To this end, we provide in this section an overview of some benchmark results on CPUs and GPUs, based on the examples shown in listings 1 and 2.

A dissection of the total computational cost of the MSFT routine reveals that time consumption of the simulation is mostly determined by the number of discrete Fourier transforms (DFTs) (one for each slice) and the target’s rendering process. The time complexity of a single DFT is given by 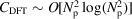 (Cooley & Tukey, 1965 ▸), where 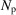 is the resolution of the output image along a single axis. The complexity for the MSFT algorithm scales linearly with the number of slices ( 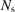 ), so that the total time complexity of the MSFT’s DFT part scales with 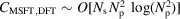 . Moreover, the time complexity of the rendering process can be roughly estimated as 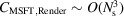 .

These considerations show that the resolution of the output image and the spatial extension of the sample, on which 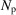 and 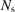 , respectively, depend, are the determining factors for the running time of a *PyScatman* simulation.

When a single shape object is given to the detector’s acquire method, the *PyScatman* module carries out the MSFT simulation differently depending on the available hardware:

(i) CPU-only systems: slice rendering is sequential, where each slice is rendered using all CPU cores in parallel. After all 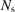 slices are rendered, all CPUs perform the DFT calculations using the embarrassingly parallel scheme.

(ii) Single NVIDIA GPU: each slice rendering and its respective DFT calculation are performed in parallel by the CUDA cores. Only one CPU is used for taking care of data preparation, inter-process communication and merging.

Therefore, if only a single shape is passed to the detector’s acquire method, as happens in listing 1, then, even in the case of a multi-GPU system, only one GPU is used, as the overhead caused by data transfers between the different GPUs’ memories would prevent a performance scaling.

However, when multiple shapes are to be simulated, as for the example presented in listing 2, multiple GPUs can speed up computation:

(i) CPU-only systems: the multiple shapes are split evenly between all available CPU cores, where, subsequently, each core takes care of performing the shape rendering and the DFT calculations.

(ii) Single NVIDIA GPU: similarly to the CPU-only case, the multiple shapes are split evenly between all available CPU cores. Each CPU then sets up the shape’s data and submits the work to the GPU, where the CUDA cores calculate the rendering and the DFTs for all slices.

(iii) Multiple NVIDIA GPUs: this is similar to the single-GPU case with the exception that the available CPUs are placed in groups where each group has an assigned GPU. For example, in an eight CPU core/four GPU system, two CPUs would share a single GPU and coordinate as in the single-GPU case.

Here, we present some benchmark results that we consider representative of real-life situations. First, note that the amount of computation, and thus the time to solution, depends on several factors (most of which can be deduced from Fig. 4 ▸):

(i) Simulation resolution: the greater the number of pixels in the output image, the greater the computational cost.

(ii) Shape extension: the greater the ratio between the sample size and the wavelength λ, the higher the number of shape voxels to be rendered. Moreover, a greater scattering angle corresponds to a greater spatial resolution, such that the number of pixels to be rendered increases accordingly with the maximum scattering angle 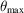 .

(iii) Shape complexity: the function that defines the shape optical properties, 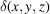 and 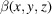 , has a non-negligible computational cost, depending on both the shape type and the input data.

Among these three aspects, the contribution of the shape complexity to the total computing time is the least straightforward to evaluate in a systematic and quantitative manner, as it is highly dependent on the shape type and the values of its parameters. For example, the SphericalHarmonics complexity is particularly low when only a few harmonic coefficients are provided as input: as the number of harmonic coefficients increases, the data preparation step, which consists of the computation of the SphericalHarmonics transform, starts to take a relevant part of the computation time. The same happens, for example, for the VolumeMap object, for which the time dedicated to data transfer has an effect on the time to solution, depending on the size of the 3D array given as input. Such a case-by-case study goes beyond the scope of this paper, and the authors encourage the reader to install the *PyScatman* module and test it for the cases of interest.

However, to give a rough idea about performance for different sample shapes, a first test is performed on the same four shape objects defined in the examples of the previous section, *i.e.* shape_el (Ellipsoid), shape_sh (Spherical­Harmonics), shape_rm (RadialMap) and shape_vm (Volume­Map), keeping the same experimental conditions and detector resolution. Here, the detector used is the MSFT one, yielding the diffraction patterns shown in Figs. 7 ▸(*e*)–7 ▸(*h*). The performance evaluation was accomplished on a workstation equipped with an Intel Core i9-9900K CPU accelerated by a GPU NVIDIA GeForce RTX 2080 Ti.

**Figure 10:**
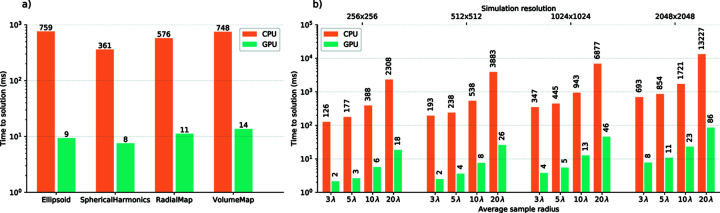
In (*a*), timing results for the shapes presented as examples in listing 1 and listing 2. The time to solution is shown in logarithmic scale versus the different shape types. In (*b*), timing results for a shape defined through the SphericalHarmonics model are given. The harmonics coefficients are the same as in the example presented in the main text, but the shape is scaled to get different average radii, indicated on the lower *x* axis in units of the wavelength λ. Results are presented for four different simulation resolutions, from 256 × 256 px up to 2048 × 2048 px, labeled on the upper *x* axis.

The simulation time is shown in Fig. 10 ▸(*a*). On the *x* axis the four different shape models are labeled. The time to solution is on the *y* axis, expressed in milliseconds on a logarithmic scale. The time shown is the execution time of a single call to the detector’s acquire method, with a single shape object given as argument and averaged over 100 repetitions to rule out statistical fluctuations. Two features are evident in the figure: first, the performance difference between the CPU time and the respective GPU time is around two orders of magnitude. Second, the time to solution depends on the shape. The first observation underlines why the *PyScatman* implementation, when executed on a GPU, enables a new kind of data analysis with the Scatman approach. The second feature, instead, is due to different shape sizes and complexities.

To quantitatively investigate the dependence of simulation time on the detector resolution and the sample’s spatial extension, a second test is presented in Fig. 10 ▸(*b*). All the timing values in this test are based upon the same sample shape rendered in Fig. 7 ▸(*b*), defined through the Spherical­Harmonics model. Here, that shape is scaled to match different average radii, 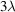 , 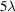 , 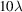 and 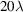 , in order to get different sample spatial extensions without varying their complexity. For each of them, two evaluations of the time to solution are performed, one running on the CPU and the other on the GPU. The whole operation is repeated for four different detector resolutions, 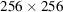 , 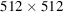 , 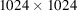 and 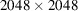 px. Here, again, the great advantage gained through the GPU computing is evident. In particular, the difference of around two orders of magnitude in the simulation time between GPU and CPU is consistently present for all the different sample sizes and the resolutions of the diffraction patterns, with the GPU still capable of performing more than ten simulations per second even in the worst, most complex case.

The presented timing results show the performance of the *PyScatman* module in the current version. The software is, however, still under development, and better timing performances are expected in future software releases thanks to a better optimization of the GPU management.

## 7. Summary

In this paper we introduced the Scatman, an approximate method to simulate wide-angle diffraction patterns from coherent and monochromatic light based on the multi-slice Fourier transform. The scientific impact of the method has already been demonstrated by previous publications that made use of the Scatman, while it was under development, to retrieve 3D morphological information on silver nanocrystals (Barke *et al.*, 2015 ▸) and helium nanodroplets (Langbehn *et al.*, 2018 ▸) from single wide-angle diffraction images.

The need for an approximate simulation tool arises from the severe limitations of the available exact methods: Mie calculations, which are fast but can be used only for highly symmetrical samples, and finite-difference time-domain or discrete dipole approximation simulations, which are computationally heavy. The Scatman was conceived to be both generic, *i.e.* capable of simulating the scattering from any kind of sample, and sufficiently fast, enabling the retrieval of the sample morphology by fitting the experimental diffraction patterns via a model fitting approach.

The mathematical foundations of the method were presented, highlighting the main approximations that make the Scatman results deviate from the exact ones. The effects of these approximations as a function of the input parameters were investigated, by comparing the Scatman simulations and the exact Mie calculations for a spherical sample. Within given bounds on the optical properties of the sample and its spatial extension, the Scatman results proved to be in quantitative agreement with exact calculations.

We presented our reference implementation of the Scatman, called *PyScatman*, which is released as open-source software with this article and is freely available online. *PyScatman*, implemented as a Python module, provides an easy interface to the user and a set of additional functionalities useful for data analysis. *PyScatman* is entirely written in C++ and makes use of state-of-the-art programming techniques to take full advantage of the most recent computing hardware, including GPU accelerators. The computational performance of *PyScatman* was briefly presented, demonstrating the possibility to perform wide-angle scattering simulations in a few to a few tens of milliseconds on consumer-level computing hardware. These computing times are well suited to the extensive use of *PyScatman* in the analysis of experimental data via forward-fitting procedures, thus opening new perspectives for coherent diffraction imaging in wide-angle scattering conditions.

The Scatman method described here is a stable and tested snapshot of its current development. Further enhancements are under study, focusing on both the physics aspect and the software implementation. In terms of software, the inclusion of additional, more sophisticated and ductile shape models is planned, along with a more efficient management of computing resources. On the physics side, the partial inclusion of secondary effects like multiple scattering, refraction and reflection is under study, extending the range of applicability of the approach to samples whose refractive index varies more strongly from unity.

The aim of the Scatman method and its software implementation is to be a reference tool for the coherent diffraction imaging community. They could also be of great interest for other scientific fields where elastic scattering of coherent radiation plays a role, like the recently growing electron diffraction imaging techniques. Moreover, the high-performance software implementation, *PyScatman*, is compatible with the increasing interest in big-data analysis and artificial intelligence (AI), and its combination with AI techniques is already in a prototyping phase.

## Supplementary Material

Supporting information. DOI: 10.1107/S1600576722008068/yr5086sup1.pdf

## Footnotes

1 A detailed explanation of every shape, along with images showing each of the axes, can be found in the official documentation under ‘Shapes’ at https://nux-group.gitlab.io/pyscatman/shapes.html.
