# Supplementary material for: The Scatman: an approximate method for fast wide-angle scattering simulations
Source: J Appl Crystallogr. 2022 Sep 14;55(Pt 5):1232–46. doi: 10.1107/S1600576722008068 (PMC9533759; doi:10.1107/S1600576722008068)
Supplement: Supplementary file 1 [file j-55-01232-sup1.pdf]

JOURNAL OF APPLIED CRYSTALLOGRAPHY

Volume 55 (2022)

Supporting information for article:

The Scatman: an approximate method for fast wide-angle scattering simulations

Alessandro Colombo, Julian Zimmermann, Bruno Langbehn, Thomas Möller, Christian Peltz, Katharina Sander, Björn Kruse, Paul Tümmler, Ingo Barke, Daniela Rupp and Thomas Fennel

The Scatman: an approximate method for fast wide-angle coherent diffraction simulations

Supplementary Material

January 21, 2022

1 Further comparisons with Mie exact results

Within the Mie framework, the intensity scattered on detector from a dielectric spherical sample surrounded by vacuum is given by:

$$I_{scat,\{||,\perp\}}(\theta, x, n) = \frac{1}{(kr)^2} i_{\{||,\perp\}}(\theta, x, n) I_{in}, \tag{S1}$$

where I_{in} is the intensity of the incoming light field, n is the refractive index inside the sphere, and $x = {}^{2\pi R}/_{\lambda}$ is called the size-parameter, with λ the wavelength of the incident radiation and R the radius of the sphere. Furthermore, the subscripts $\perp$ and $||$ denote if the incident light is polarized perpendicular or parallel to the scattering plane. The scattered irradiance per unit of incident irradiance, $i_{\{||,\perp\}}(\theta, x, n)$ is given by:

$$i_{||} = |S_2|^2 \qquad i_{\perp} = |S_1|^2 . \tag{S2}$$

S_1 and S_2 are the first and second component of the general scattering matrix [2, 1].

This section expands the comparison between the Scatman approximation and the exact Mie results to account for size dependence.

Figure S1 shows twelve contour plots in which the target's radius dependent deviations between the Scatman's and the Mie theory calculations are plotted. The figure is split into two rows and six columns, where each column addresses a different target radius (indicated by the top labels), and the two rows show different visualizations of the deviations between the Scatman's and the Mie theory calculations (indicated by the two colorbars on the right side). The top row contains contour maps of the pixel-averaged absolute relative error, which is defined as:

$$E[r, \delta, \beta] = \frac{1}{N^2} \sum_{i,j=0}^{N} \frac{\left| I_{i,j}^{\text{Scatman}}[r, \delta, \beta] - I_{i,j}^{\text{Mie}}[r, \delta, \beta] \right|}{I_{i,j}^{\text{Mie}}[r, \delta, \beta]}, \tag{S3}$$

where N is the size of the diffraction image in pixel, I is the calculated scattered intensity, and the sum runs across the whole image with index $i,\ j$. The top row, thereby, shows the magnitude of error by the Scatman routine for a given $\delta,\ \beta,\ R$ triplet in units of the Mie result. From practical experience, we refer to deviations $E[r, \delta, \beta] \lessapprox 0.1$ as quantitatively *usable*[1], and deviations $E[r, \delta, \beta] \lessapprox 0.5$ as qualitatively *usable*[2].

Since equation S3 is an average over the absolute relative error at every pixel, the information if the Scatman procedure over- or under-estimates the scattering signal is lost. For example, if the Scatman routine estimates the scattering signal for the first half of all scattering angles as half as low and then for the subsequent scattering

[1] The approximation could be used *instead* of the analytical solution for any downstream task

[2] The approximation contains all necessary features to withstand a visual comparison

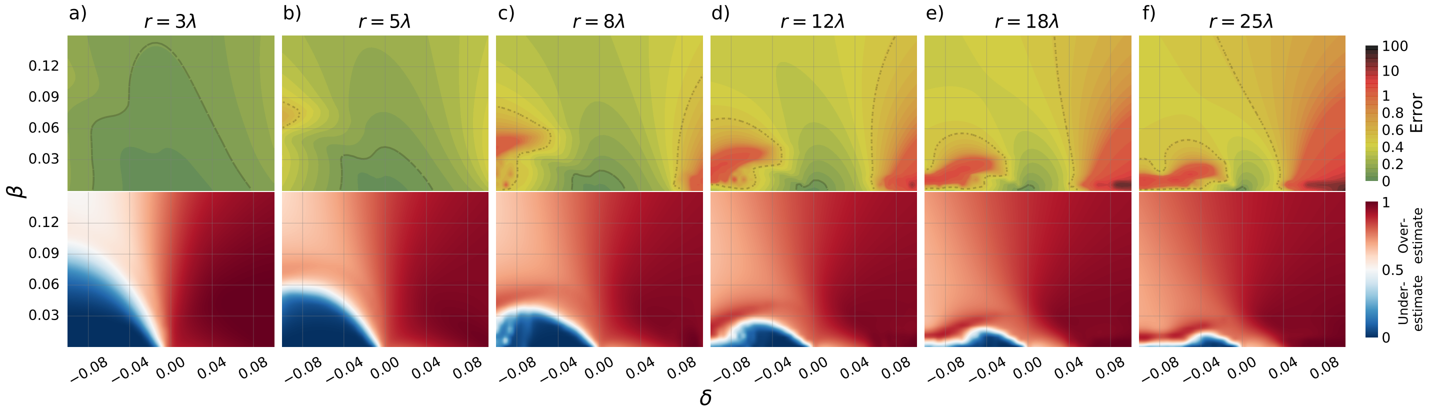

Figure S1: The target's radius dependent deviations between the Scatman's and the Mie theory calculations for different combinations of δ, β. Overall twelve contour plots are shown, where the absorption β is always located on the y-axis and the phase δ is always located on the x-axis. The figure is split into two rows and six columns, where each column shows calculations for a fixed target radius (indicated by the top labels), and the two rows show different visualizations of the deviations between the Scatman's and the Mie theory calculations (indicated by the two colorbars on the right side). The top row shows contour maps of the pixel-averaged absolute relative error where the deviation is calculated following equation S3. There, the colorbar shows a linear scale between zero and one, and a logarithmic scale between one and the maximum value. This is merely for visualization reasons, as this way the most relevant parts (between zero and one) are well discernible from the regions where the deviations are so large that it would have supplanted the region between zero and one otherwise. Two contours are displayed for each subplot: the dashed line highlights the error threshold, set to 0.1, at which the Scatman simulation can be considered in *quantitative* agreement with the exact Mie solution. Conversely, the dotted line, running over an error value equal to 0.5, highlights the boundaries of the optical properties for which the Scatman simulations are in *qualitative* agreement with Mie simulations. The bottom row's colormap shows the dissection into how much the Scatman over- or under-estimates the absolute relative error. There, a value of 0.1 means that 10 % of the pixel-averaged absolute relative error is due to overestimation and, thereby, 90 % is due to underestimation by the Scatman routine.

angles as one-and-a-half times as high as the Mie calculation, would be undiscernible from the case where the Scatman underestimates all scattering angles half as low or even where the Scatman overestimates the scattering signal at all angles one-and-a-half times as high as in the Mie calculations. All three scenarios lead to a pixel averaged absolute relative error of 0.5, yet stem from a very different simulation behavior by the Scatman.

Therefore, to resolve this ambiguity, the bottom row in Figure S1 shows the dissection of the pixel averaged absolute relative error into how much the Scatman over- or under-estimates the error. There, the three examples from above have a score of 0.5 (half of the scattering angles over- and half underestimated), 0 (all scattering angles underestimated), and 1 (all scattering angles overestimated).

The combined interpretation of both rows in Figure S1 is necessary to fully understand the scale and direction of the deviations between the Scatman routine's and the Mie theory based calculations. With that in mind, the following deductions can be made from Figure S1:

- The quality of the Scatman's approximation deteriorates globally when the radius of the target increases[3]. This is more pronounced with smaller values for β and higher values for δ
- The area with low absorption and refractive index close to unity provides the best Scatman approximation across all target's radii.
- For smaller target radii and for negative values of δ, the Scatman tends to underestimate the scattered intensity, which is more pronounced the smaller the values for β. For larger radii, the Scatman almost always overestimates the scattered intensity, only for negative values of δ and very low values for β, an underestimation remains.

[3] Or when the probing wavelength decreases

- The area at large and positive values for δ and low absorption remains the regime with the worst agreement between Mie theory and the Scatman calculations, which was already observed in Figure 4 g) of the main text (compare the solid and dashed blue lines with the red ones). This regime now grows even further when the target's radius increases as well, resulting in very strong disagreements (larger then one) between the analytical and the Scatman calculations for $\delta \gtrapprox 0.04$ at a radius of 25λ.

2 Simulation of photon statistics

The assumption at the basis of the simulation of photon statistics is that the ratio between the incoming amount of photons N_{inc} and the scattered one N_{scat} equals the ratio between the incoming power W_{in} and the scattered one W_{scat}, that is:

$$\frac{N_{scat}}{N_{in}} = \frac{W_{scat}}{W_{in}} \,. \tag{S4}$$

The incoming power can be easily computed as the integral on the x, y plane of the incoming intensity, which means:

$$W_{in} = \iint dxdy \left|\phi A_0 e^{\mathbf{i}\Phi}\right|^2 = A_0^2 \Delta x \Delta y \,, \tag{S5}$$

where Δx and Δy is the spatial extension of the incoming plane wave.

The incoming amount of photons is easily computed via:

$$N_{in} = n_{in} \Delta x \Delta y \,, \tag{S6}$$

where n_{in} is the photon density.

Finally, the scattered power can be defined as the integral over the scattered intensities:

$$\begin{aligned} W_{scat} &= \iint dq_x dq_y I(q_x, q_y) \\ &\propto \iint dq_x dq_y \left| \sum_{s=0}^{S-1} e^{-\mathbf{i}\cdot s\Delta z\, \sqrt{\mathrm{k}_0^2 - q_x{}^2 - q_y{}^2}} \mathcal{F}\Big\{ \Big[-\tilde{\delta}_s(x,y) + \mathbf{i}\tilde{\beta}_s(x,y) \Big] \, \psi_s \Big\}(q_x, q_y) \right|^2 . \end{aligned} \tag{S7}$$

However, while it was noted in the main text that it is possible to assume $\tilde{\rho}_s(x,y) \propto \Big[-\tilde{\delta}_s(x,y) + \mathbf{i}\tilde{\beta}_s(x,y) \Big]$, the proportionality constant is the one that defines the strength of the interaction between the incoming field and the sample material.

Thus, the final total amount of scattered photons is computed through:

$$N_{scat} = n_{in}\, C\, \frac{\iint dq_x dq_y I(q_x, q_y)}{A_0^2} = n_{in}^{eff} \, \frac{\iint dq_x dq_y I(q_x, q_y)}{A_0^2} \tag{S8}$$

where $n_{in}^{eff} = C\, n_{in}$ is an *effective* photon density, that has to be given as parameter to the PyScatman module. The proportionality factor C is directly connected to the *scattering efficiency* of the material, which is defined as the ratio between the scattering cross section and the geometric one.

References

[1] Craig F. Bohren and Donald R. Huffman. *Absorption and Scattering of Light by Small Particles*. Wiley, 2008.

[2] Gustav Mie. Beiträge zur Optik trüber Medien, speziell kolloidaler Metallösungen. *Ann. Phys.*, 330(3):377–445, jan 1908.
